# *In silico *evidence for the species-specific conservation of mosquito retroposons: implications as a molecular biomarker

**DOI:** 10.1186/1742-4682-6-14

**Published:** 2009-07-29

**Authors:** Wilson Byarugaba, Henry Kajumbula, Misaki Wayengera

**Affiliations:** 1 Restrizymes Biotherapeutics (U) LTD, PO Box 16606, Kampala, Uganda; 2 Dept of Postgraduate Studies and Research, Kampala International University, Western Campus, PO Box 71, Ishaka, Uganda; 3 Division of Molecular Pathology, Dept of Pathology, School of Biomedical Sciences, College of Health Sciences, Makerere University, PO Box 7072, Kampala, Uganda; 4 Division of Molecular Biology, Dept of Medical Microbiology, School of Biomedical Sciences, College of Health Sciences, Makerere University, PO Box 7072, Kampala, Uganda; 5 Dept of Immunology and Microbiology, Kampala International University, Western Campus, PO Box 71, Ishaka, Uganda

## Abstract

**Background:**

Mosquitoes are the transmissive vectors for several infectious pathogens that affect man. However, the control of mosquitoes through insecticide and pesticide spraying has proved difficult in the past. We hypothesized that, by virtue of their reported vertical inheritance among mosquitoes, group II introns – a class of small coding ribonucleic acids (scRNAs) – may form a potential species-specific biomarker. Structurally, introns are a six-moiety complex. Depending on the function of the protein encoded within the IV moiety, the highly mobile class of group II introns or retroposons is sub-divided into two: Restriction Endonuclease (REase)-like and Apurinic aPyramydinic Endonuclease (APE)-like. REase-like retroposons are thought to be the ancestors of APE retroposons. Our aim in this study was to find evidence for the highly species-specific conservation of the APE subclass of mosquito retroposons.

**Methods and Results:**

*In silico *targeted sequence alignments were conducted across a 1,779-organism genome database (1,518 bacterial, 59 archeal, 201 eukaryotic, and the human), using three mosquito retroposon sequence tags (RST) as BLASTN queries [AJ970181 and AJ90201 of *Culex pipien *origin and AJ970301 of *Anoplese sinensis *origin]. At a calibration of E = 10, A & D = 100, default filtration and a homology cut-off of >95% identity, no hits were found on any of the 1,518 bacterial genomes. Eleven (100%) and 15 (100%) hits obtained on the 201-eukaryote genome database were homologs (>95% score) of *C*.*pipien quinquefasciatus *JHB retroposons, but none of *An. sinensis*. Twenty and 221 low score (30–43% identity) spurious hits were found at flanking ends of genes and contigs in the human genome with the *C*.*pipien *and *An. sinensis *RSTs respectively. Functional and positional inference revealed these to be possible relatives of human genomic spliceosomes. We advance two models for the application of mosquito RST: as precursors for developing molecular biomarkers for mosquitoes, and as RST-specific monoclonal antibody (MAb)-DDT immunoconjugates to enhance targeted toxicity.

**Conclusion:**

We offer evidence to support the species-specific conservation of mosquito retroposons among lower taxa. Our findings suggest that retroposons may therefore constitute a unique biomarker for mosquito species that may be exploited in molecular entomology. Mosquito RST-specific MAbs may possibly permit synthesis of DDT immunoconjugates that could be used to achieve species-tailored toxicity.

## Background

### Mosquitoes are the transmissive vectors of several human infectious pathogens

Plasmodium, the causative agent of malaria, is spread by the female *anopheles *mosquito [1,2], and the nematodes *Brugia *and *Wuchereria*, which cause lymphatic filariasis (or elephantiasis), spread through the bite of the *aedes *mosquito. Among viruses, West Nile fever virus is *Culex*-mosquito borne [3-5]. Whereas the highest burdens of malaria and filariasis are found within the low income countries (LIC) of the tropics [2], West Nile Fever has been noted to cause sporadic disease in the temperate regions as well [5]. Currently, malaria is the world's 3^rd ^leading infectious cause of death globally, and lymphatic filariasis infects over 120 million people in 73 countries in Africa and India. Of the several strategies currently employed to control all three pathogens, mosquito-targeted insecticide spraying predominates [6]. Nevertheless, control of the mosquito vector through insecticide spraying has proved difficult in the past. In particular: (i) controversies have arisen surrounding the long-term toxic effects of effective agents such as DDT; (ii) there is evidence for the evolution of resistance to several insecticides and pesticides; (iii) there are notable gaps in the accurate documentation of the bionomics of mosquitoes pre- and post-spraying [6]. Addressing these three challenges is a necessary step towards the more efficient application of insecticides for controlling malaria, West Nile fever and filariasis. There are, however, no strategies in place for improving the outcomes of DDT use for mosquito control.

We conceived that one may exploit the post-genome era to address all the above problems. Our hypothesis was that group II introns – a class of small coding ribonucleic acids (scRNAs) [7,8], by virtue of their previously reported vertical inheritance among mosquitoes [9-11], may form a potential mosquito species-specific biomarker. Specifically, group II introns are a class of self-splicing and sometimes highly mobile ribonucleic acids [7]. Some have been observed to excise spontaneously from precursor messenger RNA (mRNA) and ligate their flanking exons together without the aid of a protein, as occurs in pre- and post-transcriptional nuclear mRNA intron splicing [11]. This similarity has led to the hypothesis that they may be evolutionary ancestors of spliceosomal introns, which make up about 25–35% of the human genome [12].

Structurally, all group II introns are a VI fingered (moiety) complex [7]. Retroposons, classified as Long Interspersed Nuclear Elements (LINE) of the non-Long Terminal Repeat (LTR) group [13], form a highly mobile sub-class of group II introns. This sub-class has the unique feature of encoding a reverse transcriptase (RT) open reading frame (ORF) moiety in its IV arm, which they use to insert into predefined sites at high efficacy (retrohoming) or unrelated sites at low rates (retrotransposing) [7]. Depending on the function of the major protein encoded within this moiety, retroposons may be further subdivided into Restriction Endonuclease (REase)-like and Apurinic aPyramydinic Endonuclease (APE)-like [7,9]. It is widely supposed that the REase-like retroposons are the evolutionary ancestors of the APE retroposons [10,11]. Although it is generally accepted that REase-like restroposons are inherited vertically, the inheritance of APE-like retroposons has been much debated [10,11]. While some authors provide evidence for horizontal transfer [7,12], recent evidence by Biedler and Tu [10] seems to suggest strictly vertical inheritance. Further, Crainey and colleagues [11] have employed both sub-cloning and PCR approaches to support the hypothesis that horizontal retroposon transfer does not occur or is far rarer than for other types of transposable elements.

Against the above background, this study was conducted to examine the potential of the APE subclass of retroposons as a biomarker for mosquitoes. Overall, we provide the first evidence for the species-specific conservation of mosquito retroposons.

## Results

### A. Sequence identity of mosquito APE retroposons to 1,518 bacterial and 201 eukaryotic genomes

**Figure 1:**
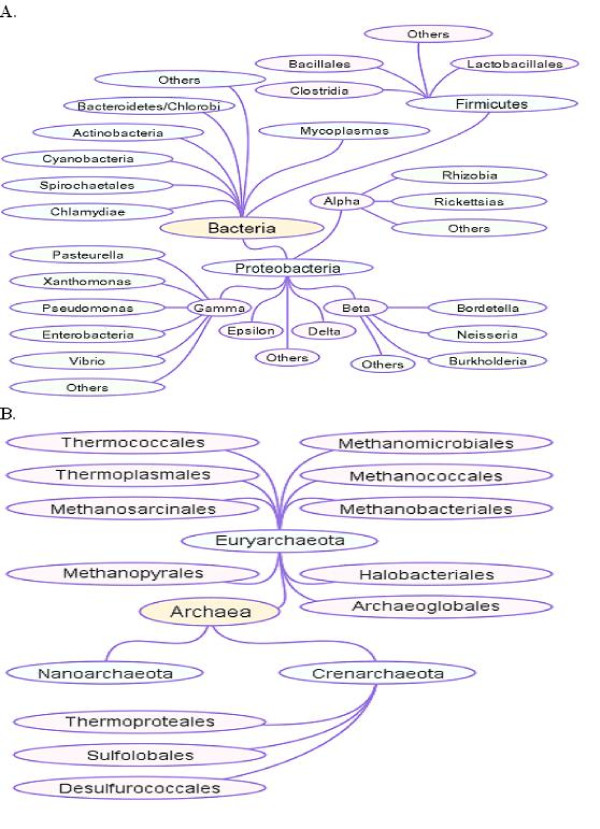
**Taxonomic tree relating the evolutionary relationship of the (A) 1, 518 bacterial and (B) 59 archael genomes searched**. The figure shows a clustered tree detailing the evolutionary relationship of the (A) 1,518 bacterial and (B) 59 archael genomes searched. This figure was obtained from and is accessible at the NCBI microbial BLAST site, URL:

The search for sequence identities between mosquito APE retroposons and genomic elements of 1,518 bacteria(see Figure 1) yielded no hits regardless of score or e-value, implying a complete absence of sequence similarity between the three mosquito retroposons and the bacterial taxa (see figure 1 for taxonomic classification). In contrast, searching the genome-wide sequence database of 201 eukaryotes for sequences identical to the three mosquito retroposons of interest revealed 11 and 15 hits corresponding respectively to *Culex pipiens *retroposon *5 Cx pip*, clone 1 and *Culex pipiens *retroposon *7 Cx pip*. All 11 hits obtained with *5 Cx pip*, clone 1 were classifiable as homologs (≥ 95% identity) (see Table 1 and [additional files 1 and 2]) of retroposons from *C. pipiens quinquefasciatus *strain JHB. Note that the C.*pipiens quinquefasciatus *strain JHB draft assembly to which these hits corresponded is part of the eukaryote genome database searched. However, there were no hits to the *An*.*sinensis *retroposon *1 An sin*, clone 5 (perhaps because the *An. sinensis *genome is currently not part of the 201-genome database).

**Table 1:** Percentage Identity of the C. *pipiens *retroposon to sequences within the 201-eukaryote genome-wide database

Contig	Identity
Culex quinquefasciatus strain JHB cont3.16735,	98
Culex quinquefasciatus strain JHB cont3.22711,	98
Culex quinquefasciatus strain JHB cont3.25671,	98
Culex quinquefasciatus strain JHB cont3.96,	98
Culex quinquefasciatus strain JHB cont3.24770,	98
Culex quinquefasciatus strain JHB cont3.26570,	98
Culex quinquefasciatus strain JHB cont3.22771,	98
Culex quinquefasciatus strain JHB cont3.15860,	98
Culex quinquefasciatus strain JHB cont3.42530,	97
Culex quinquefasciatus strain JHB cont3.39777,	97
Culex quinquefasciatus strain JHB cont3.5317,	95

Note that the query mosquito retroposon AJ970181 is a *Culex pipiens *retroposon (*5 Cx pip*, clone 1), explaining the homology observed.

### B. Identity of mosquito retroposons to human spliceosomal elements

Twenty and 221 low score (38–43) blast hits were found within the human genome corresponding to the *5 Cx pip*, clone 1 retroposon tag of *C. pipiens *and the *1 An sin*, clone 5 retroposon tag from *An. sinensis*. No hits irrespective of score were obtained with the *C*.*pipiens *retroposon tag *7 Cx pip*. Most of these spurious hits in the human genome corresponded to several sequences that may be unrelated to spliceosomal ancestors of human retroposons. For example, some of these hits corresponded to the following thirty: 12289 bp at 5' end: hypothetical protein; 1217867 bp at 3' end: chromosome 12 open reading frame 37; 8347 bp at 5' end: thymopoietin isoform beta; 34500 bp at 3' end: similar to peptidylprolyl isomerase A isoform 1; inversin isoform a; inversin isoform b; 9890 bp at 5' end: hypothetical protein LOC158405; 17561 bp at 3' end: hypothetical protein LOC58493; 79069 bp at 5' end: ACN9 homolog; 472310 bp at 3' end: tachykinin 1 isoform beta precursor; huntingtin interacting protein 1; integrin, alpha 1 precursor; 225678 bp at 3' end: embigin homolog; hexosaminidase B preproprotein; 60251 bp at 5' end: developmentally regulated protein TPO1;4025 bp at 3' end: spermatogenic leucine zipper 1; 5617 bp at 5' end: leucine-rich repeat containing 33;40193 bp at 3' end: hypothetical protein LOC84984; 12071 bp at 5' side: cysteine and glycine-rich protein 1; 140277 bp at 3' side: neuron navigator 1; G patch domain containing 2; 28533 bp at 5' side: meningioma 1; 25799 bp at 3' side: phosphatidylinositol transfer protein, beta; 3859 bp at 5' side: beta-galactoside-binding lectin precursor; 2761 bp at 3' side: nucleolar protein 12; cajalin 2 isoform a; cytokine induced protein 29 kDa; integrin, alpha 1 precursor; 227168 bp at 3' end: embigin homolog. There were also 190 more genes or contigs. However, we noted that these hits occurred most frequently at the extremities of the related genes or contigs, areas often interspersed with spliceosomes (which are designated as evolutionary ancestors of group II introns and make up 25–35% of the human genome [12] [additional file 3].

## Discussion

Our study provides the first ever comprehensive *in silco *evidence across a 1,779 genome-wide database for the highly species-specific conservation of mosquito retroposons. In the absence of a molecular biomarker for mosquitoes, entomological studies of mosquito bionomics have so far involved physical taxonomic classification. We therefore felt it necessary to identify a molecular target that may serve as a biomarker. Such a biomarker, it is envisioned, may enable mosquito speciation to be established by molecular entomology. Our work offers the first support for the hypothesis that mosquito retroposons may be exploited for that purpose. Overall, while several authors have documented the vertical inheritance of mosquitoes [10,11], most of these studies have involved too few taxa to support the concept that mosquito retroposons are highly conserved.

First, we have shown that among all three retroposons investigated, a sequence tag for one mosquito species could only be used to identify the derivative retroposon from that species. For instance, searching the entire 1,779 genome-wide nucleotide sequence database using retroposon sequence tags of the *Culex pipiens *retroposon *5 Cx pip*, clone 1 and *Culex pipiens *retroposon *7 Cx pip*, clone 3, yielded hits with contigs of *Culex pipiens quinquefasciatus *strain JHB, the sub-species of origin (see Tables 1 and [additional files 1 and 2]). This view is further supported by the finding that, since the *Anopheles sinensis *genome is not included in the genome-wide 201-eukaryote database, no hits irrespective of score were obtained with the sequence tag of *Anopheles sinensis *retroposon *1 An sin*, clone 5 as the query. Moreover, despite the presence of other related species such as *Anopheles gambiae *str. PEST and *Aedes aegypti*, none of their retroposons were identical to those of *Culex pipien *and *A*.*sinensis*. It may be argued that the power of our findings is limited by the absence from the 201-eukaryote database of more mosquito species genomes for which horizontal transfer has previously been reported [10], such as those involving (i) *Ae*.*aegypti *and *Ae*.*Albopictus*, where three cloned PCR products from *Ae*.*albopictus *are nearly identical to sequences from *Ae*.*aegypti*, (ii) *C*.*quinquefasciatus*, for which the PCR sequence groups have homology with C.*nigripalpus*, O.*atropalpus *and O.*epactius *sequences. However, Biedler and Tu [10] have recently shown that such previously-reported relationships are to be expected, since these species belong to the same species complex in which there may be introgression. Moreover, related and more comprehensive experiments based on cloning and PCR analysis of the inheritance of the Mosquito Jockey (*JM1*-Juan A and Juan C, *JM2 *and *JM3*) plus the *CR1 *clade elements have shown divergence in all groups compared, even among *Culicine *vs. *Culicine *(Cul/Cul), with increasing evolutionary distance: *Culicine *vs. *Anopheles *(Cul/An), mosquito vs. non-mosquito dipterans (Msq/dip), mosquito vs non-dipteran neoptarans (Msq/Neo) and mosquito vs. vertebrates (Msq/vert) [11].

Second, we note that among lower taxa, species-specific conservation of mosquito retroposons is common. Specifically, although several eukaryotes that contain integral mobile elements or transposable elements were part of the 201-eukaryote genome database searched, including (i) *Bombyx mori *(R2Bm element), (ii) yeast (al1 and aI2), (iii) dipterans and others [7-9,12,13], none of their respective retroposons were found to be identical to mosquito retroposons. In addition, no retroposon of bacterial origin was similar to mosquito retroposons (see Figure 1 for taxonomic tree of organisms searched bacteria and archea).

In polarity, several low score hits were found by querying the human genome with the *5 Cx pip*, clone 1 retroposon tag of *C. pipiens *and the *1 An sin*, clone 5 retroposon tag from *An. sinensis*. Note that whereas about 25–35% of the human genome [14-16] comprises Long Interspersed Nuclear Elements (LINE) or non-Long Terminal Repeats (LTR), spliceosomal elements that are considered to be ancestors of all group II introns [7,17], the low score blast hits found by aligning the three query mosquito retroposon tags against the human genome do not support consideration as homologs for which a *minimum *(>95%) identity score was set. Moreover, most have functions diverging – as shown by such examples of hits as integrin, alpha 1 precursor and spermatogenic leucine zipper 1 – from that of the six moieties of introns including reverse transcriptase or maturase activity [additional file 3] [7,17]. However, it is noticeable that most of these hits occur at extremities of the human genes or contigs, regions often flanked (interspersed) by spliceosomes. While several strategies have been used to differentiate orthologs from paralogs including the use of a protein clock and genome cross-referencing or XREFdb [18-21], we found it appropriate and easier to determine the possible relationships between mosquito retroposons and human splicesomal elements by functional and positional inference. Specifically, despite an outright absence of homology, the localization of all hits at regions occupied by splicesomes within the human genome supports prior work that identifies human genomic spliceosomal elements as possible ancestors of all group II introns [7,17]. While several bioinformatics algorithms and software with greater capacity to predict identity are available, such as space-efficient spliced alignment [22], our choice of the BLAST-N tool [23] in this study was based on its ease of access and link to the organismal genomes of interest. It is therefore likely that insignificant differences may be found when other tools are used [22]. This work, however, also serves to uniquely emphasize how simple yet reliable bioinformatics tools like BLAST may still be useful in resolving hypothetic-driven biomedical research questions and hence advancing novel drug, vaccine and diagnostic discovery. Specifically, two potential" highly innovative" applications are likely to accrue for mosquito retroposons given our findings.

First, because the foregoing evidence shows that mosquito retroposons are highly conserved within species, they may be ideal targets for research and development of mosquito-specific molecular biomarkers to employ in molecular entomology. Specifically, DNA probes or retroposon-specific monoclonal antibodies (MAbs) may be mounted on to existing platforms for the molecular characterization of pathogens, such as Polymerase Chain Reaction (PCR), DNA chips or immunohistochemistry.

**Figure 2:**
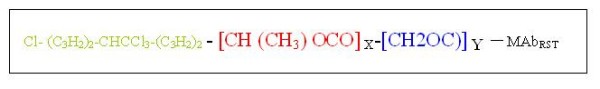
**Modeled structure of DDT and mosquito retroposon-specific monoclonal antibody (MAb) loaded nanoparticles**. The figure shows a theoretical structure of DDT and MAb_RST _loaded nanoparticles. Note that the model assumes one molecule of ingredient, although that may not be the case. The green colored formula represents a single DDT molecule whose single chain chloride ion interacts with the hydroxyls present in the lactic chain of the polyester of poly (lactic-*co*-glycolic acid) [14] commonly used to synthesize nanoparticles. The red formula bracketed × represents lactic acid, while the blue bracketed Y represents glycolic acid. Notice the availability of the hydroxyl (-OH) and free hydrogen (+H) ions at the lactic and glycolic extremities of the PLGA molecule respectively. This possibly accounts for the generality of PLGA as a solvent. MAb_RST _stands for monoclonal antibodies specific for a mosquito retroposon.

**Second**, and more speculative, is the possibility that such mosquito RST-specific MAbs may be conjugated to DDT to enhance targeted delivery of DDT to a mosquito of interest. Specifically, DDT may be conjugated to MAbs through a two step emulsion process, first incorporating DDT into the polyester PLGA, and subsequently into MAbs to form nanoparticles. The choice of design specifying the dissolution of the DDT-PLGA emulsion into MAbs is aimed at manufacturing nanoparticles coated with mosquito RST-specific MAbs. The resultant emulsion may then be allowed to nanoparticulate (precipitate) through magnetic steering as described elsewhere [24,25]. Hence, these model nanoparticles (see Figure 2) would combine DDT with MAb(s) generated from mosquito RSTs (MAb_RST_). Overall, the DDT immunoconjugate strategy is predicted to enhance the accumulation of DDT in the target rather than other organisms. We presume that the proposed DDT immunoconjugates will have the potential to eliminate the ethical controversies surrounding the cumulative toxic effects of conventional DDT. Using DDT immunoconjugates has additional advantages including the fact that, since they may ensure mosquito strain-specific toxicity, one may choose to target DDT to only those mosquito strains or species that are known vectors for pathogen(s) of public health control interest (thereby ensuring that other mosquito species not associated with disease are preserved), which is not possible with conventional unconjugated DDT. DDT immunoconjugates have another advantage in that DDT may be used at lower concentrations than are normally sprayed (dosages subtoxic to other organisms), but still attaining the levels required to kill the target species. Moreover, since DDT is bound to accumulate within the target host, resistance to DDT immunoconjugates is likely to be minimal.

While the proposed use of mosquito RST as a precursor for bioengineering mosquito-specific molecular markers is highly feasible, several concerns are apparent in the equally "highly innovative" DDT immunoconjugate model presented. First, unless novel strategies are devised that enable the MAbs to be stabilized to prolong their t1/2 on exposure to the environment, their faster biodegradation relative to DDT would render the proposed nano-constructs ineffective after a short period in the environment. However, one may still argue that, once sprayed directly into the breeding areas of mosquito larvae, namely stagnant water for the anopheles, these nanoparticles may achieve their purpose if they come quickly into contact with the larvae. It therefore becomes necessary to determine the functional t1/2 of MAbs within the DDT immunoconjugates to establish exactly how long the said nanoparticles could remain viable. Although difficult, ELISA assays may be designed to achieve such measurements, say by taking timed samples of nanoparticles exposed to harsh environments and analyzing them for binding affinity to the specified antigen (mosquito tissue sample). Also, the minimal identity among mosquito retroposons and human splicesomal elements implies that more DDT may accumulate in humans than with conventional DDT. Lethal doses of DDT among humans are however high, although comparative carcinogenic and tumogenic levels for DDT immunoconjugates would have to separately be established [6].

Second, the issue of cost is significant, unless the proposed DDT immunoconjugates are used sparingly, say by spraying directly on to the larvae within stagnant water. Specifically, since MAbs are expensive to synthesize, the cost of the proposed DDT nanoparticles would be high relative to conventional DDT. Therefore, the overall cost effectiveness of DDT immunoconjugates is debatable. In our opinion, in view of the devastating impact of diseases such as malaria on individuals and nations within malaria endemic areas, if such nanoparticles are shown by trials to have promise for eradicating malaria, it may be justifiable to invest funds in them. For instance, economists estimate that malaria accounts for approximately 40% of public health expenditure in Africa and causes an annual loss of $12 billion, or 1.3%, of the continent's gross domestic product [26,27]. This figure could be re-channeled to DDT immunoconjugates. Of course, results from actual feasibility and efficacy studies will be necessary to convince donors to decide in favor of such opinions.

## Conclusion

We offer evidence to support the species-specific conservation of mosquito retroposons. Retroposons may therefore constitute a unique biomarker for mosquito species that may be exploited in molecular entomology. The model proposing the use of mosquito RST-specific MAbs to synthesize mosquito species-tailored insecticides (DDT), however, remains speculative and highly contentious, and calls for further feasibility and effectiveness studies.

## Methods

### A. Sequence alignments with the 1,779-organism genome database

#### Design

Comparative *in silco *genomics.

#### Materials

Three retroposon sequence tags: *Culex pipiens *retroposon *5 Cx pip*, clone 1 AJ970181; *Culex pipiens *retroposon *7 Cx pip*, clone 3 AJ970201; and *An. sinensis *AJ970301), the BLAST-N tool and algorithms  and 1,779 genomes (1,518 bacterial, 59 archeal, 201 eukaryotic and the human genome build 36.2) (see Figure 1 for taxonomic tree of all bacteria and archea tested).

#### Interventions

Searching was done using the three RST (AJ970181, AJ970201 and AJ970301) as queries against the 1,778 organismal and the human genome databases by way of BLAST-N calibrated at Expect (E) = 10, Filtration (F) at Default, Description (D) and Alignment (A) at 100.

#### Measured variables

Homology was defined by a cut-off value of >95% identity. Theoretical functional inference was used to determine possible relationships among lower hits.

### B. Functional and Positional inference to define evolutionary relationship of mosquito retroposons to human spliceosomal elements

To define the exact relationship of the hits obtained by querying the *C. pipien *and *An. sinensis *retroposons against the human genome, theoretical functional inference was employed. Specifically, localization at the extremities of contigs and genes was used to infer possible spliceosomal nativity, hence evolutionary relationship.

#### Accession numbers

**Swiss Prot ***Culex pipiens *retroposon *5 Cx pip*, clone 1 AJ970181; *Culex pipiens *retroposon *7 Cx pip*, clone 3 AJ970201; and *An. sinensis *AJ970301).

## Competing interests

There are no potential sources of financial conflicts of interest to declare. BW, KH and WM are all affiliated to Restrizymes Biotherapeutics (U) LTD.

## Authors' contributions

BW and WM conceived of the study, conducted the *in silco *analyses and contributed to drafting the final manuscript. BW, WM and KH participated in data analysis and writing the final manuscript. All authors read and approved the final manuscript.

## Supplementary Material

Additional file 1 **Tabulation of score and e-values obtained by querying the *C. pipiens *retroposon **AJ970181** against the 201-eukaryote genome-wide database**. This file provides the details of scores and e-values obtained by querying the *C*.*pipiens *retroposon AJ970181 against the 201 eukaryote genome-wide database. Click here for file

Additional file 2 **Tabulation of score and e-values obtained by querying the *C. pipiens *retroposon **AJ970201** against the 201 eukaryote genome-wide database**. This file provides the details of scores and e-values obtained by querying the *C. pipiens *retroposon AJ970201 against the 201 eukaryote genome-wide database. Click here for file

Additional file 3 **Tabulation of score and e-values obtained by querying the (A) *C. pipiens *retroposon **AJ970181** and (B) *An. sinensis *retroposon **AJ970301**against the human genome and eukaryote genome-wide database**. This file provides the details of scores and e-values obtained by querying the C. *pipiens *retroposon AJ970181 and *An. sinensis *retroposon AJ970301 against the human genome and eukaryote genome-wide database. Note that the *C. pipien *retroposon AJ970201 yielded no hits regardless of score or e-value. Click here for file
