# Supplementary material for: In silico evidence for the species-specific conservation of mosquito retroposons: implications as a molecular biomarker
Source: Theor Biol Med Model. 2009 Jul 29;6:14. doi: 10.1186/1742-4682-6-14 (PMC2723080; doi:10.1186/1742-4682-6-14)
Supplement: Additional file 1 — Tabulation of score and e-values obtained by querying the C. pipiens retroposon AJ970181 against the 201-eukaryote genome-wide database. This file provides the details of scores and e-values obtained by querying the C.pipiens retroposon AJ970181 against the 201 eukaryote genome-wide database. [file 1742-4682-6-14-S1.doc]

Score E

Sequences producing significant alignments: (Bits) Value

[gb|AAWU01016735.1|](http://www.ncbi.nlm.nih.gov/entrez/query.fcgi?cmd=Retrieve&db=Nucleotide&list_uids=145456796&dopt=GenBank&RID=WPWRA923012&log$=nucltop&blast_rank=1) Culex quinquefasciatus strain JHB cont3.16...  [675](http://www.ncbi.nlm.nih.gov/blast/Blast.cgi#145456796%23145456796) 0.0

[gb|AAWU01022711.1|](http://www.ncbi.nlm.nih.gov/entrez/query.fcgi?cmd=Retrieve&db=Nucleotide&list_uids=145450352&dopt=GenBank&RID=WPWRA923012&log$=nucltop&blast_rank=2) Culex quinquefasciatus strain JHB cont3.22...  [675](http://www.ncbi.nlm.nih.gov/blast/Blast.cgi#145450352%23145450352) 0.0

[gb|AAWU01025671.1|](http://www.ncbi.nlm.nih.gov/entrez/query.fcgi?cmd=Retrieve&db=Nucleotide&list_uids=145447384&dopt=GenBank&RID=WPWRA923012&log$=nucltop&blast_rank=3) Culex quinquefasciatus strain JHB cont3.25...  [675](http://www.ncbi.nlm.nih.gov/blast/Blast.cgi#145447384%23145447384) 0.0

[gb|AAWU01039777.1|](http://www.ncbi.nlm.nih.gov/entrez/query.fcgi?cmd=Retrieve&db=Nucleotide&list_uids=145433169&dopt=GenBank&RID=WPWRA923012&log$=nucltop&blast_rank=4) Culex quinquefasciatus strain JHB cont3.39...  [671](http://www.ncbi.nlm.nih.gov/blast/Blast.cgi#145433169%23145433169) 0.0

[gb|AAWU01000096.1|](http://www.ncbi.nlm.nih.gov/entrez/query.fcgi?cmd=Retrieve&db=Nucleotide&list_uids=145473437&dopt=GenBank&RID=WPWRA923012&log$=nucltop&blast_rank=5) Culex quinquefasciatus strain JHB cont3.96...  [669](http://www.ncbi.nlm.nih.gov/blast/Blast.cgi#145473437%23145473437) 0.0

[gb|AAWU01024770.1|](http://www.ncbi.nlm.nih.gov/entrez/query.fcgi?cmd=Retrieve&db=Nucleotide&list_uids=145448285&dopt=GenBank&RID=WPWRA923012&log$=nucltop&blast_rank=6) Culex quinquefasciatus strain JHB cont3.24...  [669](http://www.ncbi.nlm.nih.gov/blast/Blast.cgi#145448285%23145448285) 0.0

[gb|AAWU01026570.1|](http://www.ncbi.nlm.nih.gov/entrez/query.fcgi?cmd=Retrieve&db=Nucleotide&list_uids=145446485&dopt=GenBank&RID=WPWRA923012&log$=nucltop&blast_rank=7) Culex quinquefasciatus strain JHB cont3.26...  [669](http://www.ncbi.nlm.nih.gov/blast/Blast.cgi#145446485%23145446485) 0.0

[gb|AAWU01022771.1|](http://www.ncbi.nlm.nih.gov/entrez/query.fcgi?cmd=Retrieve&db=Nucleotide&list_uids=145450292&dopt=GenBank&RID=WPWRA923012&log$=nucltop&blast_rank=8) Culex quinquefasciatus strain JHB cont3.22...  [507](http://www.ncbi.nlm.nih.gov/blast/Blast.cgi#145450292%23145450292) 2e-140

[gb|AAWU01015860.1|](http://www.ncbi.nlm.nih.gov/entrez/query.fcgi?cmd=Retrieve&db=Nucleotide&list_uids=145457671&dopt=GenBank&RID=WPWRA923012&log$=nucltop&blast_rank=9) Culex quinquefasciatus strain JHB cont3.15...  [387](http://www.ncbi.nlm.nih.gov/blast/Blast.cgi#145457671%23145457671) 3e-104

[gb|AAWU01005317.1|](http://www.ncbi.nlm.nih.gov/entrez/query.fcgi?cmd=Retrieve&db=Nucleotide&list_uids=145468214&dopt=GenBank&RID=WPWRA923012&log$=nucltop&blast_rank=10) Culex quinquefasciatus strain JHB cont3.53...  [147](http://www.ncbi.nlm.nih.gov/blast/Blast.cgi#145468214%23145468214) 6e-32

[gb|AAWU01042530.1|](http://www.ncbi.nlm.nih.gov/entrez/query.fcgi?cmd=Retrieve&db=Nucleotide&list_uids=145430365&dopt=GenBank&RID=WPWRA923012&log$=nucltop&blast_rank=11) Culex quinquefasciatus strain JHB cont3.42... [76.8](http://www.ncbi.nlm.nih.gov/blast/Blast.cgi#145430365%23145430365) 8e-11
