# Supplementary material for: In silico evidence for the species-specific conservation of mosquito retroposons: implications as a molecular biomarker
Source: Theor Biol Med Model. 2009 Jul 29;6:14. doi: 10.1186/1742-4682-6-14 (PMC2723080; doi:10.1186/1742-4682-6-14)
Supplement: Additional file 2 — Tabulation of score and e-values obtained by querying the C. pipiens retroposon AJ970201 against the 201 eukaryote genome-wide database. This file provides the details of scores and e-values obtained by querying the C. pipiens retroposon AJ970201 against the 201 eukaryote genome-wide database. [file 1742-4682-6-14-S2.doc]

Score E

Sequences producing significant alignments: (Bits) Value

[gb|AAWU01000812.1|](http://www.ncbi.nlm.nih.gov/entrez/query.fcgi?cmd=Retrieve&db=Nucleotide&list_uids=145472721&dopt=GenBank&RID=JW9KVHK011) Culex pipiens quinquefasciatus strain JHB ...  [686](http://www.ncbi.nlm.nih.gov/blast/Blast.cgi#145472721%23145472721) 0.0

[gb|AAWU01010584.1|](http://www.ncbi.nlm.nih.gov/entrez/query.fcgi?cmd=Retrieve&db=Nucleotide&list_uids=145462947&dopt=GenBank&RID=JW9KVHK011) Culex pipiens quinquefasciatus strain JHB ...  [686](http://www.ncbi.nlm.nih.gov/blast/Blast.cgi#145462947%23145462947) 0.0

[gb|AAWU01014406.1|](http://www.ncbi.nlm.nih.gov/entrez/query.fcgi?cmd=Retrieve&db=Nucleotide&list_uids=145459125&dopt=GenBank&RID=JW9KVHK011) Culex pipiens quinquefasciatus strain JHB ...  [686](http://www.ncbi.nlm.nih.gov/blast/Blast.cgi#145459125%23145459125) 0.0

[gb|AAWU01022460.1|](http://www.ncbi.nlm.nih.gov/entrez/query.fcgi?cmd=Retrieve&db=Nucleotide&list_uids=145450603&dopt=GenBank&RID=JW9KVHK011) Culex pipiens quinquefasciatus strain JHB ...  [686](http://www.ncbi.nlm.nih.gov/blast/Blast.cgi#145450603%23145450603) 0.0

[gb|AAWU01030710.1|](http://www.ncbi.nlm.nih.gov/entrez/query.fcgi?cmd=Retrieve&db=Nucleotide&list_uids=145442332&dopt=GenBank&RID=JW9KVHK011) Culex pipiens quinquefasciatus strain JHB ...  [686](http://www.ncbi.nlm.nih.gov/blast/Blast.cgi#145442332%23145442332) 0.0

[gb|AAWU01032707.1|](http://www.ncbi.nlm.nih.gov/entrez/query.fcgi?cmd=Retrieve&db=Nucleotide&list_uids=145440239&dopt=GenBank&RID=JW9KVHK011) Culex pipiens quinquefasciatus strain JHB ...  [686](http://www.ncbi.nlm.nih.gov/blast/Blast.cgi#145440239%23145440239) 0.0

[gb|AAWU01034550.1|](http://www.ncbi.nlm.nih.gov/entrez/query.fcgi?cmd=Retrieve&db=Nucleotide&list_uids=145438396&dopt=GenBank&RID=JW9KVHK011) Culex pipiens quinquefasciatus strain JHB ...  [680](http://www.ncbi.nlm.nih.gov/blast/Blast.cgi#145438396%23145438396) 0.0

[gb|AAWU01029781.1|](http://www.ncbi.nlm.nih.gov/entrez/query.fcgi?cmd=Retrieve&db=Nucleotide&list_uids=145443274&dopt=GenBank&RID=JW9KVHK011) Culex pipiens quinquefasciatus strain JHB ...  [675](http://www.ncbi.nlm.nih.gov/blast/Blast.cgi#145443274%23145443274) 0.0

[gb|AAWU01005871.1|](http://www.ncbi.nlm.nih.gov/entrez/query.fcgi?cmd=Retrieve&db=Nucleotide&list_uids=145467660&dopt=GenBank&RID=JW9KVHK011) Culex pipiens quinquefasciatus strain JHB ...  [669](http://www.ncbi.nlm.nih.gov/blast/Blast.cgi#145467660%23145467660) 0.0

[gb|AAWU01015412.1|](http://www.ncbi.nlm.nih.gov/entrez/query.fcgi?cmd=Retrieve&db=Nucleotide&list_uids=145458119&dopt=GenBank&RID=JW9KVHK011) Culex pipiens quinquefasciatus strain JHB ...  [669](http://www.ncbi.nlm.nih.gov/blast/Blast.cgi#145458119%23145458119) 0.0

[gb|AAWU01042189.1|](http://www.ncbi.nlm.nih.gov/entrez/query.fcgi?cmd=Retrieve&db=Nucleotide&list_uids=145430706&dopt=GenBank&RID=JW9KVHK011) Culex pipiens quinquefasciatus strain JHB ...  [669](http://www.ncbi.nlm.nih.gov/blast/Blast.cgi#145430706%23145430706) 0.0

[gb|AAWU01010176.1|](http://www.ncbi.nlm.nih.gov/entrez/query.fcgi?cmd=Retrieve&db=Nucleotide&list_uids=145463355&dopt=GenBank&RID=JW9KVHK011) Culex pipiens quinquefasciatus strain JHB ...  [664](http://www.ncbi.nlm.nih.gov/blast/Blast.cgi#145463355%23145463355) 0.0

[gb|AAWU01036696.1|](http://www.ncbi.nlm.nih.gov/entrez/query.fcgi?cmd=Retrieve&db=Nucleotide&list_uids=145436250&dopt=GenBank&RID=JW9KVHK011) Culex pipiens quinquefasciatus strain JHB ...  [649](http://www.ncbi.nlm.nih.gov/blast/Blast.cgi#145436250%23145436250) 0.0

[gb|AAWU01026391.1|](http://www.ncbi.nlm.nih.gov/entrez/query.fcgi?cmd=Retrieve&db=Nucleotide&list_uids=145446664&dopt=GenBank&RID=JW9KVHK011) Culex pipiens quinquefasciatus strain JHB ...  [641](http://www.ncbi.nlm.nih.gov/blast/Blast.cgi#145446664%23145446664) 2e-180

[gb|AAWU01022330.1|](http://www.ncbi.nlm.nih.gov/entrez/query.fcgi?cmd=Retrieve&db=Nucleotide&list_uids=145450733&dopt=GenBank&RID=JW9KVHK011) Culex pipiens quinquefasciatus strain JHB ...  [449](http://www.ncbi.nlm.nih.gov/blast/Blast.cgi#145450733%23145450733) 1e-122
