# Supplementary material for: In silico evidence for the species-specific conservation of mosquito retroposons: implications as a molecular biomarker
Source: Theor Biol Med Model. 2009 Jul 29;6:14. doi: 10.1186/1742-4682-6-14 (PMC2723080; doi:10.1186/1742-4682-6-14)
Supplement: Additional file 3 — Tabulation of score and e-values obtained by querying the (A) C. pipiens retroposon AJ970181 and (B) An. sinensis retroposon AJ970301against the human genome and eukaryote genome-wide database. This file provides the details of scores and e-values obtained by querying the C. pipiens retroposon AJ970181 and An. sinensis retroposon AJ970301 against the human genome and eukaryote genome-wide database. Note that the C. pipien retroposon AJ970201 yielded no hits regardless of score or e-value. [file 1742-4682-6-14-S3.doc]

A.

Score E

Sequences producing significant alignments: (Bits) Value

[ref|NT_004487.18|Hs1_4644](http://www.ncbi.nlm.nih.gov/mapview/maps.cgi?maps=blast_set&db=all_contig&na=1&gnl=ref|NT_004487.18|Hs1_4644&gi=88943682&term=88943682%5Bgi%5D&taxid=9606&RID=JWA3SX7N011&QUERY_NUMBER=1) Homo sapiens chromosome 1 genomic cont [42.1](http://www.ncbi.nlm.nih.gov/blast/Blast.cgi#88943682%2388943682) 0.46

[ref|NW_926128.1|HsCraAADB02_53](http://www.ncbi.nlm.nih.gov/mapview/maps.cgi?maps=blast_set&db=all_contig&na=1&gnl=ref|NW_926128.1|HsCraAADB02_53&gi=88952696&term=88952696%5Bgi%5D&taxid=9606&RID=JWA3SX7N011&QUERY_NUMBER=1) Homo sapiens chromosome 1 geno... [42.1](http://www.ncbi.nlm.nih.gov/blast/Blast.cgi#88952696%2388952696) 0.46

[ref|NT_029419.11|Hs12_29578](http://www.ncbi.nlm.nih.gov/mapview/maps.cgi?maps=blast_set&db=all_contig&na=1&gnl=ref|NT_029419.11|Hs12_29578&gi=89035619&term=89035619%5Bgi%5D&taxid=9606&RID=JWA3SX7N011&QUERY_NUMBER=1) Homo sapiens chromosome 12 genomic c [40.1](http://www.ncbi.nlm.nih.gov/blast/Blast.cgi#89035619%2389035619) 1.8

[ref|NW_925395.1|HsCraAADB02_464](http://www.ncbi.nlm.nih.gov/mapview/maps.cgi?maps=blast_set&db=all_contig&na=1&gnl=ref|NW_925395.1|HsCraAADB02_464&gi=89036563&term=89036563%5Bgi%5D&taxid=9606&RID=JWA3SX7N011&QUERY_NUMBER=1) Homo sapiens chromosome 12 ge... [40.1](http://www.ncbi.nlm.nih.gov/blast/Blast.cgi#89036563%2389036563) 1.8

[ref|NT_011651.16|HsX_11808](http://www.ncbi.nlm.nih.gov/mapview/maps.cgi?maps=blast_set&db=all_contig&na=1&gnl=ref|NT_011651.16|HsX_11808&gi=89060003&term=89060003%5Bgi%5D&taxid=9606&RID=JWA3SX7N011&QUERY_NUMBER=1) Homo sapiens chromosome X genomic con [38.2](http://www.ncbi.nlm.nih.gov/blast/Blast.cgi#89060003%2389060003) 7.2

[ref|NT_011512.10|Hs21_11669](http://www.ncbi.nlm.nih.gov/mapview/maps.cgi?maps=blast_set&db=all_contig&na=1&gnl=ref|NT_011512.10|Hs21_11669&gi=51475294&term=51475294%5Bgi%5D&taxid=9606&RID=JWA3SX7N011&QUERY_NUMBER=1) Homo sapiens chromosome 21 genomic c [38.2](http://www.ncbi.nlm.nih.gov/blast/Blast.cgi#51475294%2351475294) 7.2

[ref|NT_025004.13|Hs18_25160](http://www.ncbi.nlm.nih.gov/mapview/maps.cgi?maps=blast_set&db=all_contig&na=1&gnl=ref|NT_025004.13|Hs18_25160&gi=29823169&term=29823169%5Bgi%5D&taxid=9606&RID=JWA3SX7N011&QUERY_NUMBER=1) Homo sapiens chromosome 18 genomic c [38.2](http://www.ncbi.nlm.nih.gov/blast/Blast.cgi#29823169%2329823169) 7.2

[ref|NT_008583.16|Hs10_8740](http://www.ncbi.nlm.nih.gov/mapview/maps.cgi?maps=blast_set&db=all_contig&na=1&gnl=ref|NT_008583.16|Hs10_8740&gi=37551286&term=37551286%5Bgi%5D&taxid=9606&RID=JWA3SX7N011&QUERY_NUMBER=1) Homo sapiens chromosome 10 genomic co [38.2](http://www.ncbi.nlm.nih.gov/blast/Blast.cgi#37551286%2337551286) 7.2

[ref|NT_007592.14|Hs6_7749](http://www.ncbi.nlm.nih.gov/mapview/maps.cgi?maps=blast_set&db=all_contig&na=1&gnl=ref|NT_007592.14|Hs6_7749&gi=51465675&term=51465675%5Bgi%5D&taxid=9606&RID=JWA3SX7N011&QUERY_NUMBER=1) Homo sapiens chromosome 6 genomic cont [38.2](http://www.ncbi.nlm.nih.gov/blast/Blast.cgi#51465675%2351465675) 7.2

[ref|NT_023133.12|Hs5_23289](http://www.ncbi.nlm.nih.gov/mapview/maps.cgi?maps=blast_set&db=all_contig&na=1&gnl=ref|NT_023133.12|Hs5_23289&gi=51464637&term=51464637%5Bgi%5D&taxid=9606&RID=JWA3SX7N011&QUERY_NUMBER=1) Homo sapiens chromosome 5 genomic con [38.2](http://www.ncbi.nlm.nih.gov/blast/Blast.cgi#51464637%2351464637) 7.2

[ref|NT_005612.15|Hs3_5769](http://www.ncbi.nlm.nih.gov/mapview/maps.cgi?maps=blast_set&db=all_contig&na=1&gnl=ref|NT_005612.15|Hs3_5769&gi=88966845&term=88966845%5Bgi%5D&taxid=9606&RID=JWA3SX7N011&QUERY_NUMBER=1) Homo sapiens chromosome 3 genomic cont [38.2](http://www.ncbi.nlm.nih.gov/blast/Blast.cgi#88966845%2388966845) 7.2

[ref|NW_927715.1|HsCraAADB02_686](http://www.ncbi.nlm.nih.gov/mapview/maps.cgi?maps=blast_set&db=all_contig&na=1&gnl=ref|NW_927715.1|HsCraAADB02_686&gi=89060930&term=89060930%5Bgi%5D&taxid=9606&RID=JWA3SX7N011&QUERY_NUMBER=1) Homo sapiens chromosome X gen... [38.2](http://www.ncbi.nlm.nih.gov/blast/Blast.cgi#89060930%2389060930) 7.2

[ref|NW_927384.1|HsCraAADB02_643](http://www.ncbi.nlm.nih.gov/mapview/maps.cgi?maps=blast_set&db=all_contig&na=1&gnl=ref|NW_927384.1|HsCraAADB02_643&gi=89058398&term=89058398%5Bgi%5D&taxid=9606&RID=JWA3SX7N011&QUERY_NUMBER=1) Homo sapiens chromosome 21 ge... [38.2](http://www.ncbi.nlm.nih.gov/blast/Blast.cgi#89058398%2389058398) 7.2

[ref|NW_927129.1|HsCraAADB02_620](http://www.ncbi.nlm.nih.gov/mapview/maps.cgi?maps=blast_set&db=all_contig&na=1&gnl=ref|NW_927129.1|HsCraAADB02_620&gi=89047539&term=89047539%5Bgi%5D&taxid=9606&RID=JWA3SX7N011&QUERY_NUMBER=1) Homo sapiens chromosome 18 ge... [38.2](http://www.ncbi.nlm.nih.gov/blast/Blast.cgi#89047539%2389047539) 7.2

[ref|NW_924796.1|HsCraAADB02_410](http://www.ncbi.nlm.nih.gov/mapview/maps.cgi?maps=blast_set&db=all_contig&na=1&gnl=ref|NW_924796.1|HsCraAADB02_410&gi=89032038&term=89032038%5Bgi%5D&taxid=9606&RID=JWA3SX7N011&QUERY_NUMBER=1) Homo sapiens chromosome 10 ge... [38.2](http://www.ncbi.nlm.nih.gov/blast/Blast.cgi#89032038%2389032038) 7.2

[ref|NW_923073.1|HsCraAADB02_255](http://www.ncbi.nlm.nih.gov/mapview/maps.cgi?maps=blast_set&db=all_contig&na=1&gnl=ref|NW_923073.1|HsCraAADB02_255&gi=88999178&term=88999178%5Bgi%5D&taxid=9606&RID=JWA3SX7N011&QUERY_NUMBER=1) Homo sapiens chromosome 6 gen... [38.2](http://www.ncbi.nlm.nih.gov/blast/Blast.cgi#88999178%2388999178) 7.2

[ref|NW_922818.1|HsCraAADB02_232](http://www.ncbi.nlm.nih.gov/mapview/maps.cgi?maps=blast_set&db=all_contig&na=1&gnl=ref|NW_922818.1|HsCraAADB02_232&gi=88990170&term=88990170%5Bgi%5D&taxid=9606&RID=JWA3SX7N011&QUERY_NUMBER=1) Homo sapiens chromosome 5 gen... [38.2](http://www.ncbi.nlm.nih.gov/blast/Blast.cgi#88990170%2388990170) 7.2

### [**ref|NW_921807.1|HsCraAADB02_141**](http://www.ncbi.nlm.nih.gov/mapview/maps.cgi?maps=blast_set&db=all_contig&na=1&gnl=ref|NW_921807.1|HsCraAADB02_141&gi=88971630&term=88971630%5Bgi%5D&taxid=9606&RID=JWA3SX7N011&QUERY_NUMBER=1) Homo sapiens chromosome 3 gen... **[38.2](http://www.ncbi.nlm.nih.gov/blast/Blast.cgi#88971630%2388971630)** 7.2

B.

Score E

Sequences producing significant alignments: (Bits) Value

[ref|NT_011520.11|Hs22_11677](http://www.ncbi.nlm.nih.gov/mapview/maps.cgi?maps=blast_set&db=all_contig&na=1&gnl=ref|NT_011520.11|Hs22_11677&gi=89059027&term=89059027%5Bgi%5D&taxid=9606&RID=JWC9C717011&QUERY_NUMBER=1) Homo sapiens chromosome 22 genomic c [40.1](http://www.ncbi.nlm.nih.gov/blast/Blast.cgi#89059027%2389059027) 1.8

[ref|NT_019546.15|Hs12_19702](http://www.ncbi.nlm.nih.gov/mapview/maps.cgi?maps=blast_set&db=all_contig&na=1&gnl=ref|NT_019546.15|Hs12_19702&gi=29802923&term=29802923%5Bgi%5D&taxid=9606&RID=JWC9C717011&QUERY_NUMBER=1) Homo sapiens chromosome 12 genomic c [40.1](http://www.ncbi.nlm.nih.gov/blast/Blast.cgi#29802923%2329802923) 1.8

[ref|NT_008470.18|Hs9_8627](http://www.ncbi.nlm.nih.gov/mapview/maps.cgi?maps=blast_set&db=all_contig&na=1&gnl=ref|NT_008470.18|Hs9_8627&gi=89030004&term=89030004%5Bgi%5D&taxid=9606&RID=JWC9C717011&QUERY_NUMBER=1) Homo sapiens chromosome 9 genomic cont [40.1](http://www.ncbi.nlm.nih.gov/blast/Blast.cgi#89030004%2389030004) 1.8

[ref|NT_007933.14|Hs7_8090](http://www.ncbi.nlm.nih.gov/mapview/maps.cgi?maps=blast_set&db=all_contig&na=1&gnl=ref|NT_007933.14|Hs7_8090&gi=51493052&term=51493052%5Bgi%5D&taxid=9606&RID=JWC9C717011&QUERY_NUMBER=1) Homo sapiens chromosome 7 genomic cont [40.1](http://www.ncbi.nlm.nih.gov/blast/Blast.cgi#51493052%2351493052) 1.8

[ref|NT_006713.14|Hs5_6870](http://www.ncbi.nlm.nih.gov/mapview/maps.cgi?maps=blast_set&db=all_contig&na=1&gnl=ref|NT_006713.14|Hs5_6870&gi=51465008&term=51465008%5Bgi%5D&taxid=9606&RID=JWC9C717011&QUERY_NUMBER=1) Homo sapiens chromosome 5 genomic cont [40.1](http://www.ncbi.nlm.nih.gov/blast/Blast.cgi#51465008%2351465008) 1.8

[ref|NT_029928.12|Hs3_30183](http://www.ncbi.nlm.nih.gov/mapview/maps.cgi?maps=blast_set&db=all_contig&na=1&gnl=ref|NT_029928.12|Hs3_30183&gi=51464066&term=51464066%5Bgi%5D&taxid=9606&RID=JWC9C717011&QUERY_NUMBER=1) Homo sapiens chromosome 3 genomic con [40.1](http://www.ncbi.nlm.nih.gov/blast/Blast.cgi#51464066%2351464066) 1.8

[ref|NT_004487.18|Hs1_4644](http://www.ncbi.nlm.nih.gov/mapview/maps.cgi?maps=blast_set&db=all_contig&na=1&gnl=ref|NT_004487.18|Hs1_4644&gi=88943682&term=88943682%5Bgi%5D&taxid=9606&RID=JWC9C717011&QUERY_NUMBER=1) Homo sapiens chromosome 1 genomic cont [40.1](http://www.ncbi.nlm.nih.gov/blast/Blast.cgi#88943682%2388943682) 1.8

[ref|NT_021877.18|Hs1_22033](http://www.ncbi.nlm.nih.gov/mapview/maps.cgi?maps=blast_set&db=all_contig&na=1&gnl=ref|NT_021877.18|Hs1_22033&gi=88943807&term=88943807%5Bgi%5D&taxid=9606&RID=JWC9C717011&QUERY_NUMBER=1) Homo sapiens chromosome 1 genomic con [40.1](http://www.ncbi.nlm.nih.gov/blast/Blast.cgi#88943807%2388943807) 1.8

[ref|NW_927628.1|HsCraAADB02_665](http://www.ncbi.nlm.nih.gov/mapview/maps.cgi?maps=blast_set&db=all_contig&na=1&gnl=ref|NW_927628.1|HsCraAADB02_665&gi=89059359&term=89059359%5Bgi%5D&taxid=9606&RID=JWC9C717011&QUERY_NUMBER=1) Homo sapiens chromosome 22 ge... [40.1](http://www.ncbi.nlm.nih.gov/blast/Blast.cgi#89059359%2389059359) 1.8

[ref|NW_925395.1|HsCraAADB02_464](http://www.ncbi.nlm.nih.gov/mapview/maps.cgi?maps=blast_set&db=all_contig&na=1&gnl=ref|NW_925395.1|HsCraAADB02_464&gi=89036563&term=89036563%5Bgi%5D&taxid=9606&RID=JWC9C717011&QUERY_NUMBER=1) Homo sapiens chromosome 12 ge... [40.1](http://www.ncbi.nlm.nih.gov/blast/Blast.cgi#89036563%2389036563) 1.8

[ref|NW_924539.1|HsCraAADB02_387](http://www.ncbi.nlm.nih.gov/mapview/maps.cgi?maps=blast_set&db=all_contig&na=1&gnl=ref|NW_924539.1|HsCraAADB02_387&gi=89030529&term=89030529%5Bgi%5D&taxid=9606&RID=JWC9C717011&QUERY_NUMBER=1) Homo sapiens chromosome 9 gen... [40.1](http://www.ncbi.nlm.nih.gov/blast/Blast.cgi#89030529%2389030529) 1.8

[ref|NT_079595.2|Hs7_79660](http://www.ncbi.nlm.nih.gov/mapview/maps.cgi?maps=blast_set&db=all_contig&na=1&gnl=ref|NT_079595.2|Hs7_79660&gi=89027401&term=89027401%5Bgi%5D&taxid=9606&RID=JWC9C717011&QUERY_NUMBER=1) Homo sapiens chromosome 7 genomic c... [40.1](http://www.ncbi.nlm.nih.gov/blast/Blast.cgi#89027401%2389027401) 1.8

[ref|NW_923574.1|HsCraAADB02_300](http://www.ncbi.nlm.nih.gov/mapview/maps.cgi?maps=blast_set&db=all_contig&na=1&gnl=ref|NW_923574.1|HsCraAADB02_300&gi=89026252&term=89026252%5Bgi%5D&taxid=9606&RID=JWC9C717011&QUERY_NUMBER=1) Homo sapiens chromosome 7 gen... [40.1](http://www.ncbi.nlm.nih.gov/blast/Blast.cgi#89026252%2389026252) 1.8

[ref|NW_922607.1|HsCraAADB02_213](http://www.ncbi.nlm.nih.gov/mapview/maps.cgi?maps=blast_set&db=all_contig&na=1&gnl=ref|NW_922607.1|HsCraAADB02_213&gi=88987778&term=88987778%5Bgi%5D&taxid=9606&RID=JWC9C717011&QUERY_NUMBER=1) Homo sapiens chromosome 5 gen... [40.1](http://www.ncbi.nlm.nih.gov/blast/Blast.cgi#88987778%2388987778) 1.8

[ref|NW_921873.1|HsCraAADB02_147](http://www.ncbi.nlm.nih.gov/mapview/maps.cgi?maps=blast_set&db=all_contig&na=1&gnl=ref|NW_921873.1|HsCraAADB02_147&gi=88971850&term=88971850%5Bgi%5D&taxid=9606&RID=JWC9C717011&QUERY_NUMBER=1) Homo sapiens chromosome 3 gen... [40.1](http://www.ncbi.nlm.nih.gov/blast/Blast.cgi#88971850%2388971850) 1.8

[ref|NW_926794.1|HsCraAADB02_59](http://www.ncbi.nlm.nih.gov/mapview/maps.cgi?maps=blast_set&db=all_contig&na=1&gnl=ref|NW_926794.1|HsCraAADB02_59&gi=88952819&term=88952819%5Bgi%5D&taxid=9606&RID=JWC9C717011&QUERY_NUMBER=1) Homo sapiens chromosome 1 geno... [40.1](http://www.ncbi.nlm.nih.gov/blast/Blast.cgi#88952819%2388952819) 1.8

[ref|NW_926128.1|HsCraAADB02_53](http://www.ncbi.nlm.nih.gov/mapview/maps.cgi?maps=blast_set&db=all_contig&na=1&gnl=ref|NW_926128.1|HsCraAADB02_53&gi=88952696&term=88952696%5Bgi%5D&taxid=9606&RID=JWC9C717011&QUERY_NUMBER=1) Homo sapiens chromosome 1 geno... [40.1](http://www.ncbi.nlm.nih.gov/blast/Blast.cgi#88952696%2388952696) 1.8

[ref|NT_011875.11|HsY_12032](http://www.ncbi.nlm.nih.gov/mapview/maps.cgi?maps=blast_set&db=all_contig&na=1&gnl=ref|NT_011875.11|HsY_12032&gi=51477752&term=51477752%5Bgi%5D&taxid=9606&RID=JWC9C717011&QUERY_NUMBER=1) Homo sapiens chromosome Y genomic con [38.2](http://www.ncbi.nlm.nih.gov/blast/Blast.cgi#51477752%2351477752) 7.3

[ref|NT_011669.16|HsX_11826](http://www.ncbi.nlm.nih.gov/mapview/maps.cgi?maps=blast_set&db=all_contig&na=1&gnl=ref|NT_011669.16|HsX_11826&gi=89059864&term=89059864%5Bgi%5D&taxid=9606&RID=JWC9C717011&QUERY_NUMBER=1) Homo sapiens chromosome X genomic con [38.2](http://www.ncbi.nlm.nih.gov/blast/Blast.cgi#89059864%2389059864) 7.3

[ref|NT_010498.15|Hs16_10655](http://www.ncbi.nlm.nih.gov/mapview/maps.cgi?maps=blast_set&db=all_contig&na=1&gnl=ref|NT_010498.15|Hs16_10655&gi=51473102&term=51473102%5Bgi%5D&taxid=9606&RID=JWC9C717011&QUERY_NUMBER=1) Homo sapiens chromosome 16 genomic c [38.2](http://www.ncbi.nlm.nih.gov/blast/Blast.cgi#51473102%2351473102) 7.3

[ref|NT_035325.6|Hs15_35487](http://www.ncbi.nlm.nih.gov/mapview/maps.cgi?maps=blast_set&db=all_contig&na=1&gnl=ref|NT_035325.6|Hs15_35487&gi=51472610&term=51472610%5Bgi%5D&taxid=9606&RID=JWC9C717011&QUERY_NUMBER=1) Homo sapiens chromosome 15 genomic co [38.2](http://www.ncbi.nlm.nih.gov/blast/Blast.cgi#51472610%2351472610) 7.3

[ref|NT_030059.12|Hs10_30314](http://www.ncbi.nlm.nih.gov/mapview/maps.cgi?maps=blast_set&db=all_contig&na=1&gnl=ref|NT_030059.12|Hs10_30314&gi=51467897&term=51467897%5Bgi%5D&taxid=9606&RID=JWC9C717011&QUERY_NUMBER=1) Homo sapiens chromosome 10 genomic c [38.2](http://www.ncbi.nlm.nih.gov/blast/Blast.cgi#51467897%2351467897) 7.3

[ref|NT_005612.15|Hs3_5769](http://www.ncbi.nlm.nih.gov/mapview/maps.cgi?maps=blast_set&db=all_contig&na=1&gnl=ref|NT_005612.15|Hs3_5769&gi=88966845&term=88966845%5Bgi%5D&taxid=9606&RID=JWC9C717011&QUERY_NUMBER=1) Homo sapiens chromosome 3 genomic cont [38.2](http://www.ncbi.nlm.nih.gov/blast/Blast.cgi#88966845%2388966845) 7.3

[ref|NT_005403.16|Hs2_5560](http://www.ncbi.nlm.nih.gov/mapview/maps.cgi?maps=blast_set&db=all_contig&na=1&gnl=ref|NT_005403.16|Hs2_5560&gi=88954065&term=88954065%5Bgi%5D&taxid=9606&RID=JWC9C717011&QUERY_NUMBER=1) Homo sapiens chromosome 2 genomic cont [38.2](http://www.ncbi.nlm.nih.gov/blast/Blast.cgi#88954065%2388954065) 7.3

[ref|NT_022184.14|Hs2_22340](http://www.ncbi.nlm.nih.gov/mapview/maps.cgi?maps=blast_set&db=all_contig&na=1&gnl=ref|NT_022184.14|Hs2_22340&gi=51460714&term=51460714%5Bgi%5D&taxid=9606&RID=JWC9C717011&QUERY_NUMBER=1) Homo sapiens chromosome 2 genomic con [38.2](http://www.ncbi.nlm.nih.gov/blast/Blast.cgi#51460714%2351460714) 7.3

[ref|NT_022135.15|Hs2_22291](http://www.ncbi.nlm.nih.gov/mapview/maps.cgi?maps=blast_set&db=all_contig&na=1&gnl=ref|NT_022135.15|Hs2_22291&gi=88953723&term=88953723%5Bgi%5D&taxid=9606&RID=JWC9C717011&QUERY_NUMBER=1) Homo sapiens chromosome 2 genomic con [38.2](http://www.ncbi.nlm.nih.gov/blast/Blast.cgi#88953723%2388953723) 7.3

[ref|NW_927716.1|HsCraAADB02_687](http://www.ncbi.nlm.nih.gov/mapview/maps.cgi?maps=blast_set&db=all_contig&na=1&gnl=ref|NW_927716.1|HsCraAADB02_687&gi=89060947&term=89060947%5Bgi%5D&taxid=9606&RID=JWC9C717011&QUERY_NUMBER=1) Homo sapiens chromosome X gen... [38.2](http://www.ncbi.nlm.nih.gov/blast/Blast.cgi#89060947%2389060947) 7.3

[ref|NW_927715.1|HsCraAADB02_686](http://www.ncbi.nlm.nih.gov/mapview/maps.cgi?maps=blast_set&db=all_contig&na=1&gnl=ref|NW_927715.1|HsCraAADB02_686&gi=89060930&term=89060930%5Bgi%5D&taxid=9606&RID=JWC9C717011&QUERY_NUMBER=1) Homo sapiens chromosome X gen... [38.2](http://www.ncbi.nlm.nih.gov/blast/Blast.cgi#89060930%2389060930) 7.3

[ref|NW_927713.1|HsCraAADB02_684](http://www.ncbi.nlm.nih.gov/mapview/maps.cgi?maps=blast_set&db=all_contig&na=1&gnl=ref|NW_927713.1|HsCraAADB02_684&gi=89060901&term=89060901%5Bgi%5D&taxid=9606&RID=JWC9C717011&QUERY_NUMBER=1) Homo sapiens chromosome X gen... [38.2](http://www.ncbi.nlm.nih.gov/blast/Blast.cgi#89060901%2389060901) 7.3

[ref|NW_927711.1|HsCraAADB02_682](http://www.ncbi.nlm.nih.gov/mapview/maps.cgi?maps=blast_set&db=all_contig&na=1&gnl=ref|NW_927711.1|HsCraAADB02_682&gi=89060842&term=89060842%5Bgi%5D&taxid=9606&RID=JWC9C717011&QUERY_NUMBER=1) Homo sapiens chromosome X gen... [38.2](http://www.ncbi.nlm.nih.gov/blast/Blast.cgi#89060842%2389060842) 7.3

[ref|NW_927706.1|HsCraAADB02_678](http://www.ncbi.nlm.nih.gov/mapview/maps.cgi?maps=blast_set&db=all_contig&na=1&gnl=ref|NW_927706.1|HsCraAADB02_678&gi=89060788&term=89060788%5Bgi%5D&taxid=9606&RID=JWC9C717011&QUERY_NUMBER=1) Homo sapiens chromosome X gen... [38.2](http://www.ncbi.nlm.nih.gov/blast/Blast.cgi#89060788%2389060788) 7.3

[ref|NW_927702.1|HsCraAADB02_674](http://www.ncbi.nlm.nih.gov/mapview/maps.cgi?maps=blast_set&db=all_contig&na=1&gnl=ref|NW_927702.1|HsCraAADB02_674&gi=89060744&term=89060744%5Bgi%5D&taxid=9606&RID=JWC9C717011&QUERY_NUMBER=1) Homo sapiens chromosome X gen... [38.2](http://www.ncbi.nlm.nih.gov/blast/Blast.cgi#89060744%2389060744) 7.3

[ref|NW_927700.1|HsCraAADB02_672](http://www.ncbi.nlm.nih.gov/mapview/maps.cgi?maps=blast_set&db=all_contig&na=1&gnl=ref|NW_927700.1|HsCraAADB02_672&gi=89060678&term=89060678%5Bgi%5D&taxid=9606&RID=JWC9C717011&QUERY_NUMBER=1) Homo sapiens chromosome X gen... [38.2](http://www.ncbi.nlm.nih.gov/blast/Blast.cgi#89060678%2389060678) 7.3

[ref|NW_927339.1|HsCraAADB02_639](http://www.ncbi.nlm.nih.gov/mapview/maps.cgi?maps=blast_set&db=all_contig&na=1&gnl=ref|NW_927339.1|HsCraAADB02_639&gi=89058110&term=89058110%5Bgi%5D&taxid=9606&RID=JWC9C717011&QUERY_NUMBER=1) Homo sapiens chromosome 20 ge... [38.2](http://www.ncbi.nlm.nih.gov/blast/Blast.cgi#89058110%2389058110) 7.3

[ref|NW_927317.1|HsCraAADB02_637](http://www.ncbi.nlm.nih.gov/mapview/maps.cgi?maps=blast_set&db=all_contig&na=1&gnl=ref|NW_927317.1|HsCraAADB02_637&gi=89058024&term=89058024%5Bgi%5D&taxid=9606&RID=JWC9C717011&QUERY_NUMBER=1) Homo sapiens chromosome 20 ge... [38.2](http://www.ncbi.nlm.nih.gov/blast/Blast.cgi#89058024%2389058024) 7.3

[ref|NW_927195.1|HsCraAADB02_626](http://www.ncbi.nlm.nih.gov/mapview/maps.cgi?maps=blast_set&db=all_contig&na=1&gnl=ref|NW_927195.1|HsCraAADB02_626&gi=89057343&term=89057343%5Bgi%5D&taxid=9606&RID=JWC9C717011&QUERY_NUMBER=1) Homo sapiens chromosome 19 ge... [38.2](http://www.ncbi.nlm.nih.gov/blast/Blast.cgi#89057343%2389057343) 7.3

[ref|NW_927173.1|HsCraAADB02_624](http://www.ncbi.nlm.nih.gov/mapview/maps.cgi?maps=blast_set&db=all_contig&na=1&gnl=ref|NW_927173.1|HsCraAADB02_624&gi=89057118&term=89057118%5Bgi%5D&taxid=9606&RID=JWC9C717011&QUERY_NUMBER=1) Homo sapiens chromosome 19 ge... [38.2](http://www.ncbi.nlm.nih.gov/blast/Blast.cgi#89057118%2389057118) 7.3

[ref|NW_927206.1|HsCraAADB02_627](http://www.ncbi.nlm.nih.gov/mapview/maps.cgi?maps=blast_set&db=all_contig&na=1&gnl=ref|NW_927206.1|HsCraAADB02_627&gi=89057433&term=89057433%5Bgi%5D&taxid=9606&RID=JWC9C717011&QUERY_NUMBER=1) Homo sapiens chromosome 19 ge... [38.2](http://www.ncbi.nlm.nih.gov/blast/Blast.cgi#89057433%2389057433) 7.3

[ref|NW_927129.1|HsCraAADB02_620](http://www.ncbi.nlm.nih.gov/mapview/maps.cgi?maps=blast_set&db=all_contig&na=1&gnl=ref|NW_927129.1|HsCraAADB02_620&gi=89047539&term=89047539%5Bgi%5D&taxid=9606&RID=JWC9C717011&QUERY_NUMBER=1) Homo sapiens chromosome 18 ge... [38.2](http://www.ncbi.nlm.nih.gov/blast/Blast.cgi#89047539%2389047539) 7.3

[ref|NW_927106.1|HsCraAADB02_618](http://www.ncbi.nlm.nih.gov/mapview/maps.cgi?maps=blast_set&db=all_contig&na=1&gnl=ref|NW_927106.1|HsCraAADB02_618&gi=89047489&term=89047489%5Bgi%5D&taxid=9606&RID=JWC9C717011&QUERY_NUMBER=1) Homo sapiens chromosome 18 ge... [38.2](http://www.ncbi.nlm.nih.gov/blast/Blast.cgi#89047489%2389047489) 7.3

[ref|NW_927095.1|HsCraAADB02_617](http://www.ncbi.nlm.nih.gov/mapview/maps.cgi?maps=blast_set&db=all_contig&na=1&gnl=ref|NW_927095.1|HsCraAADB02_617&gi=89047430&term=89047430%5Bgi%5D&taxid=9606&RID=JWC9C717011&QUERY_NUMBER=1) Homo sapiens chromosome 18 ge... [38.2](http://www.ncbi.nlm.nih.gov/blast/Blast.cgi#89047430%2389047430) 7.3

[ref|NW_926940.1|HsCraAADB02_603](http://www.ncbi.nlm.nih.gov/mapview/maps.cgi?maps=blast_set&db=all_contig&na=1&gnl=ref|NW_926940.1|HsCraAADB02_603&gi=89047334&term=89047334%5Bgi%5D&taxid=9606&RID=JWC9C717011&QUERY_NUMBER=1) Homo sapiens chromosome 18 ge... [38.2](http://www.ncbi.nlm.nih.gov/blast/Blast.cgi#89047334%2389047334) 7.3

[ref|NW_926918.1|HsCraAADB02_601](http://www.ncbi.nlm.nih.gov/mapview/maps.cgi?maps=blast_set&db=all_contig&na=1&gnl=ref|NW_926918.1|HsCraAADB02_601&gi=89043126&term=89043126%5Bgi%5D&taxid=9606&RID=JWC9C717011&QUERY_NUMBER=1) Homo sapiens chromosome 17 ge... [38.2](http://www.ncbi.nlm.nih.gov/blast/Blast.cgi#89043126%2389043126) 7.3

[ref|NW_926462.1|HsCraAADB02_560](http://www.ncbi.nlm.nih.gov/mapview/maps.cgi?maps=blast_set&db=all_contig&na=1&gnl=ref|NW_926462.1|HsCraAADB02_560&gi=89041005&term=89041005%5Bgi%5D&taxid=9606&RID=JWC9C717011&QUERY_NUMBER=1) Homo sapiens chromosome 16 ge... [38.2](http://www.ncbi.nlm.nih.gov/blast/Blast.cgi#89041005%2389041005) 7.3

[ref|NW_926528.1|HsCraAADB02_566](http://www.ncbi.nlm.nih.gov/mapview/maps.cgi?maps=blast_set&db=all_contig&na=1&gnl=ref|NW_926528.1|HsCraAADB02_566&gi=89041077&term=89041077%5Bgi%5D&taxid=9606&RID=JWC9C717011&QUERY_NUMBER=1) Homo sapiens chromosome 16 ge... [38.2](http://www.ncbi.nlm.nih.gov/blast/Blast.cgi#89041077%2389041077) 7.3

[ref|NW_925907.1|HsCraAADB02_510](http://www.ncbi.nlm.nih.gov/mapview/maps.cgi?maps=blast_set&db=all_contig&na=1&gnl=ref|NW_925907.1|HsCraAADB02_510&gi=89039206&term=89039206%5Bgi%5D&taxid=9606&RID=JWC9C717011&QUERY_NUMBER=1) Homo sapiens chromosome 15 ge... [38.2](http://www.ncbi.nlm.nih.gov/blast/Blast.cgi#89039206%2389039206) 7.3

[ref|NW_925884.1|HsCraAADB02_508](http://www.ncbi.nlm.nih.gov/mapview/maps.cgi?maps=blast_set&db=all_contig&na=1&gnl=ref|NW_925884.1|HsCraAADB02_508&gi=89039160&term=89039160%5Bgi%5D&taxid=9606&RID=JWC9C717011&QUERY_NUMBER=1) Homo sapiens chromosome 15 ge... [38.2](http://www.ncbi.nlm.nih.gov/blast/Blast.cgi#89039160%2389039160) 7.3

[ref|NW_925840.1|HsCraAADB02_504](http://www.ncbi.nlm.nih.gov/mapview/maps.cgi?maps=blast_set&db=all_contig&na=1&gnl=ref|NW_925840.1|HsCraAADB02_504&gi=89038972&term=89038972%5Bgi%5D&taxid=9606&RID=JWC9C717011&QUERY_NUMBER=1) Homo sapiens chromosome 15 ge... [38.2](http://www.ncbi.nlm.nih.gov/blast/Blast.cgi#89038972%2389038972) 7.3

[ref|NW_925940.1|HsCraAADB02_513](http://www.ncbi.nlm.nih.gov/mapview/maps.cgi?maps=blast_set&db=all_contig&na=1&gnl=ref|NW_925940.1|HsCraAADB02_513&gi=89039352&term=89039352%5Bgi%5D&taxid=9606&RID=JWC9C717011&QUERY_NUMBER=1) Homo sapiens chromosome 15 ge... [38.2](http://www.ncbi.nlm.nih.gov/blast/Blast.cgi#89039352%2389039352) 7.3

[ref|NW_925561.1|HsCraAADB02_479](http://www.ncbi.nlm.nih.gov/mapview/maps.cgi?maps=blast_set&db=all_contig&na=1&gnl=ref|NW_925561.1|HsCraAADB02_479&gi=89037929&term=89037929%5Bgi%5D&taxid=9606&RID=JWC9C717011&QUERY_NUMBER=1) Homo sapiens chromosome 14 ge... [38.2](http://www.ncbi.nlm.nih.gov/blast/Blast.cgi#89037929%2389037929) 7.3

[ref|NW_925539.1|HsCraAADB02_477](http://www.ncbi.nlm.nih.gov/mapview/maps.cgi?maps=blast_set&db=all_contig&na=1&gnl=ref|NW_925539.1|HsCraAADB02_477&gi=89037680&term=89037680%5Bgi%5D&taxid=9606&RID=JWC9C717011&QUERY_NUMBER=1) Homo sapiens chromosome 14 ge... [38.2](http://www.ncbi.nlm.nih.gov/blast/Blast.cgi#89037680%2389037680) 7.3

[ref|NW_925473.1|HsCraAADB02_471](http://www.ncbi.nlm.nih.gov/mapview/maps.cgi?maps=blast_set&db=all_contig&na=1&gnl=ref|NW_925473.1|HsCraAADB02_471&gi=89037061&term=89037061%5Bgi%5D&taxid=9606&RID=JWC9C717011&QUERY_NUMBER=1) Homo sapiens chromosome 13 ge... [38.2](http://www.ncbi.nlm.nih.gov/blast/Blast.cgi#89037061%2389037061) 7.3

[ref|NW_925517.1|HsCraAADB02_475](http://www.ncbi.nlm.nih.gov/mapview/maps.cgi?maps=blast_set&db=all_contig&na=1&gnl=ref|NW_925517.1|HsCraAADB02_475&gi=89037217&term=89037217%5Bgi%5D&taxid=9606&RID=JWC9C717011&QUERY_NUMBER=1) Homo sapiens chromosome 13 ge... [38.2](http://www.ncbi.nlm.nih.gov/blast/Blast.cgi#89037217%2389037217) 7.3

[ref|NW_925328.1|HsCraAADB02_458](http://www.ncbi.nlm.nih.gov/mapview/maps.cgi?maps=blast_set&db=all_contig&na=1&gnl=ref|NW_925328.1|HsCraAADB02_458&gi=89036076&term=89036076%5Bgi%5D&taxid=9606&RID=JWC9C717011&QUERY_NUMBER=1) Homo sapiens chromosome 12 ge... [38.2](http://www.ncbi.nlm.nih.gov/blast/Blast.cgi#89036076%2389036076) 7.3

[ref|NW_925295.1|HsCraAADB02_455](http://www.ncbi.nlm.nih.gov/mapview/maps.cgi?maps=blast_set&db=all_contig&na=1&gnl=ref|NW_925295.1|HsCraAADB02_455&gi=89035948&term=89035948%5Bgi%5D&taxid=9606&RID=JWC9C717011&QUERY_NUMBER=1) Homo sapiens chromosome 12 ge... [38.2](http://www.ncbi.nlm.nih.gov/blast/Blast.cgi#89035948%2389035948) 7.3

[ref|NW_925284.1|HsCraAADB02_454](http://www.ncbi.nlm.nih.gov/mapview/maps.cgi?maps=blast_set&db=all_contig&na=1&gnl=ref|NW_925284.1|HsCraAADB02_454&gi=89035870&term=89035870%5Bgi%5D&taxid=9606&RID=JWC9C717011&QUERY_NUMBER=1) Homo sapiens chromosome 12 ge... [38.2](http://www.ncbi.nlm.nih.gov/blast/Blast.cgi#89035870%2389035870) 7.3

[ref|NW_925106.1|HsCraAADB02_438](http://www.ncbi.nlm.nih.gov/mapview/maps.cgi?maps=blast_set&db=all_contig&na=1&gnl=ref|NW_925106.1|HsCraAADB02_438&gi=89035018&term=89035018%5Bgi%5D&taxid=9606&RID=JWC9C717011&QUERY_NUMBER=1) Homo sapiens chromosome 11 ge... [38.2](http://www.ncbi.nlm.nih.gov/blast/Blast.cgi#89035018%2389035018) 7.3

[ref|NW_925006.1|HsCraAADB02_429](http://www.ncbi.nlm.nih.gov/mapview/maps.cgi?maps=blast_set&db=all_contig&na=1&gnl=ref|NW_925006.1|HsCraAADB02_429&gi=89034692&term=89034692%5Bgi%5D&taxid=9606&RID=JWC9C717011&QUERY_NUMBER=1) Homo sapiens chromosome 11 ge... [38.2](http://www.ncbi.nlm.nih.gov/blast/Blast.cgi#89034692%2389034692) 7.3

[ref|NW_925173.1|HsCraAADB02_444](http://www.ncbi.nlm.nih.gov/mapview/maps.cgi?maps=blast_set&db=all_contig&na=1&gnl=ref|NW_925173.1|HsCraAADB02_444&gi=89035221&term=89035221%5Bgi%5D&taxid=9606&RID=JWC9C717011&QUERY_NUMBER=1) Homo sapiens chromosome 11 ge... [38.2](http://www.ncbi.nlm.nih.gov/blast/Blast.cgi#89035221%2389035221) 7.3

[ref|NW_924862.1|HsCraAADB02_416](http://www.ncbi.nlm.nih.gov/mapview/maps.cgi?maps=blast_set&db=all_contig&na=1&gnl=ref|NW_924862.1|HsCraAADB02_416&gi=89033516&term=89033516%5Bgi%5D&taxid=9606&RID=JWC9C717011&QUERY_NUMBER=1) Homo sapiens chromosome 10 ge... [38.2](http://www.ncbi.nlm.nih.gov/blast/Blast.cgi#89033516%2389033516) 7.3

[ref|NW_924796.1|HsCraAADB02_410](http://www.ncbi.nlm.nih.gov/mapview/maps.cgi?maps=blast_set&db=all_contig&na=1&gnl=ref|NW_924796.1|HsCraAADB02_410&gi=89032038&term=89032038%5Bgi%5D&taxid=9606&RID=JWC9C717011&QUERY_NUMBER=1) Homo sapiens chromosome 10 ge... [38.2](http://www.ncbi.nlm.nih.gov/blast/Blast.cgi#89032038%2389032038) 7.3

[ref|NW_924696.1|HsCraAADB02_401](http://www.ncbi.nlm.nih.gov/mapview/maps.cgi?maps=blast_set&db=all_contig&na=1&gnl=ref|NW_924696.1|HsCraAADB02_401&gi=89031955&term=89031955%5Bgi%5D&taxid=9606&RID=JWC9C717011&QUERY_NUMBER=1) Homo sapiens chromosome 10 ge... [38.2](http://www.ncbi.nlm.nih.gov/blast/Blast.cgi#89031955%2389031955) 7.3

[ref|NW_924584.1|HsCraAADB02_391](http://www.ncbi.nlm.nih.gov/mapview/maps.cgi?maps=blast_set&db=all_contig&na=1&gnl=ref|NW_924584.1|HsCraAADB02_391&gi=89031869&term=89031869%5Bgi%5D&taxid=9606&RID=JWC9C717011&QUERY_NUMBER=1) Homo sapiens chromosome 10 ge... [38.2](http://www.ncbi.nlm.nih.gov/blast/Blast.cgi#89031869%2389031869) 7.3

[ref|NW_924884.1|HsCraAADB02_418](http://www.ncbi.nlm.nih.gov/mapview/maps.cgi?maps=blast_set&db=all_contig&na=1&gnl=ref|NW_924884.1|HsCraAADB02_418&gi=89033689&term=89033689%5Bgi%5D&taxid=9606&RID=JWC9C717011&QUERY_NUMBER=1) Homo sapiens chromosome 10 ge... [38.2](http://www.ncbi.nlm.nih.gov/blast/Blast.cgi#89033689%2389033689) 7.3

[ref|NW_924062.1|HsCraAADB02_344](http://www.ncbi.nlm.nih.gov/mapview/maps.cgi?maps=blast_set&db=all_contig&na=1&gnl=ref|NW_924062.1|HsCraAADB02_344&gi=89030318&term=89030318%5Bgi%5D&taxid=9606&RID=JWC9C717011&QUERY_NUMBER=1) Homo sapiens chromosome 9 gen... [38.2](http://www.ncbi.nlm.nih.gov/blast/Blast.cgi#89030318%2389030318) 7.3

[ref|NW_924484.1|HsCraAADB02_382](http://www.ncbi.nlm.nih.gov/mapview/maps.cgi?maps=blast_set&db=all_contig&na=1&gnl=ref|NW_924484.1|HsCraAADB02_382&gi=89030471&term=89030471%5Bgi%5D&taxid=9606&RID=JWC9C717011&QUERY_NUMBER=1) Homo sapiens chromosome 9 gen... [38.2](http://www.ncbi.nlm.nih.gov/blast/Blast.cgi#89030471%2389030471) 7.3

[ref|NW_924450.1|HsCraAADB02_379](http://www.ncbi.nlm.nih.gov/mapview/maps.cgi?maps=blast_set&db=all_contig&na=1&gnl=ref|NW_924450.1|HsCraAADB02_379&gi=89030365&term=89030365%5Bgi%5D&taxid=9606&RID=JWC9C717011&QUERY_NUMBER=1) Homo sapiens chromosome 9 gen... [38.2](http://www.ncbi.nlm.nih.gov/blast/Blast.cgi#89030365%2389030365) 7.3

[ref|NW_923929.1|HsCraAADB02_332](http://www.ncbi.nlm.nih.gov/mapview/maps.cgi?maps=blast_set&db=all_contig&na=1&gnl=ref|NW_923929.1|HsCraAADB02_332&gi=89028792&term=89028792%5Bgi%5D&taxid=9606&RID=JWC9C717011&QUERY_NUMBER=1) Homo sapiens chromosome 8 gen... [38.2](http://www.ncbi.nlm.nih.gov/blast/Blast.cgi#89028792%2389028792) 7.3

[ref|NW_923907.1|HsCraAADB02_330](http://www.ncbi.nlm.nih.gov/mapview/maps.cgi?maps=blast_set&db=all_contig&na=1&gnl=ref|NW_923907.1|HsCraAADB02_330&gi=89028628&term=89028628%5Bgi%5D&taxid=9606&RID=JWC9C717011&QUERY_NUMBER=1) Homo sapiens chromosome 8 gen... [38.2](http://www.ncbi.nlm.nih.gov/blast/Blast.cgi#89028628%2389028628) 7.3

[ref|NW_923873.1|HsCraAADB02_327](http://www.ncbi.nlm.nih.gov/mapview/maps.cgi?maps=blast_set&db=all_contig&na=1&gnl=ref|NW_923873.1|HsCraAADB02_327&gi=89028523&term=89028523%5Bgi%5D&taxid=9606&RID=JWC9C717011&QUERY_NUMBER=1) Homo sapiens chromosome 8 gen... [38.2](http://www.ncbi.nlm.nih.gov/blast/Blast.cgi#89028523%2389028523) 7.3

[ref|NW_923984.1|HsCraAADB02_337](http://www.ncbi.nlm.nih.gov/mapview/maps.cgi?maps=blast_set&db=all_contig&na=1&gnl=ref|NW_923984.1|HsCraAADB02_337&gi=89028938&term=89028938%5Bgi%5D&taxid=9606&RID=JWC9C717011&QUERY_NUMBER=1) Homo sapiens chromosome 8 gen... [38.2](http://www.ncbi.nlm.nih.gov/blast/Blast.cgi#89028938%2389028938) 7.3

[ref|NW_923240.1|HsCraAADB02_270](http://www.ncbi.nlm.nih.gov/mapview/maps.cgi?maps=blast_set&db=all_contig&na=1&gnl=ref|NW_923240.1|HsCraAADB02_270&gi=89025910&term=89025910%5Bgi%5D&taxid=9606&RID=JWC9C717011&QUERY_NUMBER=1) Homo sapiens chromosome 7 gen... [38.2](http://www.ncbi.nlm.nih.gov/blast/Blast.cgi#89025910%2389025910) 7.3

[ref|NT_079593.2|Hs7_79658](http://www.ncbi.nlm.nih.gov/mapview/maps.cgi?maps=blast_set&db=all_contig&na=1&gnl=ref|NT_079593.2|Hs7_79658&gi=89027233&term=89027233%5Bgi%5D&taxid=9606&RID=JWC9C717011&QUERY_NUMBER=1) Homo sapiens chromosome 7 genomic c... [38.2](http://www.ncbi.nlm.nih.gov/blast/Blast.cgi#89027233%2389027233) 7.3

[ref|NW_923640.1|HsCraAADB02_306](http://www.ncbi.nlm.nih.gov/mapview/maps.cgi?maps=blast_set&db=all_contig&na=1&gnl=ref|NW_923640.1|HsCraAADB02_306&gi=89026473&term=89026473%5Bgi%5D&taxid=9606&RID=JWC9C717011&QUERY_NUMBER=1) Homo sapiens chromosome 7 gen... [38.2](http://www.ncbi.nlm.nih.gov/blast/Blast.cgi#89026473%2389026473) 7.3

[ref|NW_923206.1|HsCraAADB02_267](http://www.ncbi.nlm.nih.gov/mapview/maps.cgi?maps=blast_set&db=all_contig&na=1&gnl=ref|NW_923206.1|HsCraAADB02_267&gi=89025729&term=89025729%5Bgi%5D&taxid=9606&RID=JWC9C717011&QUERY_NUMBER=1) Homo sapiens chromosome 7 gen... [38.2](http://www.ncbi.nlm.nih.gov/blast/Blast.cgi#89025729%2389025729) 7.3

[ref|NT_079592.2|Hs7_79657](http://www.ncbi.nlm.nih.gov/mapview/maps.cgi?maps=blast_set&db=all_contig&na=1&gnl=ref|NT_079592.2|Hs7_79657&gi=89026958&term=89026958%5Bgi%5D&taxid=9606&RID=JWC9C717011&QUERY_NUMBER=1) Homo sapiens chromosome 7 genomic c... [38.2](http://www.ncbi.nlm.nih.gov/blast/Blast.cgi#89026958%2389026958) 7.3

[ref|NT_079596.2|Hs7_79661](http://www.ncbi.nlm.nih.gov/mapview/maps.cgi?maps=blast_set&db=all_contig&na=1&gnl=ref|NT_079596.2|Hs7_79661&gi=89027696&term=89027696%5Bgi%5D&taxid=9606&RID=JWC9C717011&QUERY_NUMBER=1) Homo sapiens chromosome 7 genomic c... [38.2](http://www.ncbi.nlm.nih.gov/blast/Blast.cgi#89027696%2389027696) 7.3

[ref|NW_923484.1|HsCraAADB02_292](http://www.ncbi.nlm.nih.gov/mapview/maps.cgi?maps=blast_set&db=all_contig&na=1&gnl=ref|NW_923484.1|HsCraAADB02_292&gi=89026127&term=89026127%5Bgi%5D&taxid=9606&RID=JWC9C717011&QUERY_NUMBER=1) Homo sapiens chromosome 7 gen... [38.2](http://www.ncbi.nlm.nih.gov/blast/Blast.cgi#89026127%2389026127) 7.3

[ref|NW_923417.1|HsCraAADB02_286](http://www.ncbi.nlm.nih.gov/mapview/maps.cgi?maps=blast_set&db=all_contig&na=1&gnl=ref|NW_923417.1|HsCraAADB02_286&gi=89026092&term=89026092%5Bgi%5D&taxid=9606&RID=JWC9C717011&QUERY_NUMBER=1) Homo sapiens chromosome 7 gen... [38.2](http://www.ncbi.nlm.nih.gov/blast/Blast.cgi#89026092%2389026092) 7.3

[ref|NW_923184.1|HsCraAADB02_265](http://www.ncbi.nlm.nih.gov/mapview/maps.cgi?maps=blast_set&db=all_contig&na=1&gnl=ref|NW_923184.1|HsCraAADB02_265&gi=88999564&term=88999564%5Bgi%5D&taxid=9606&RID=JWC9C717011&QUERY_NUMBER=1) Homo sapiens chromosome 6 gen... [38.2](http://www.ncbi.nlm.nih.gov/blast/Blast.cgi#88999564%2388999564) 7.3

[ref|NW_922984.1|HsCraAADB02_247](http://www.ncbi.nlm.nih.gov/mapview/maps.cgi?maps=blast_set&db=all_contig&na=1&gnl=ref|NW_922984.1|HsCraAADB02_247&gi=88998930&term=88998930%5Bgi%5D&taxid=9606&RID=JWC9C717011&QUERY_NUMBER=1) Homo sapiens chromosome 6 gen... [38.2](http://www.ncbi.nlm.nih.gov/blast/Blast.cgi#88998930%2388998930) 7.3

[ref|NT_113893.1|Hs6_111612](http://www.ncbi.nlm.nih.gov/mapview/maps.cgi?maps=blast_set&db=all_contig&na=1&gnl=ref|NT_113893.1|Hs6_111612&gi=88998766&term=88998766%5Bgi%5D&taxid=9606&RID=JWC9C717011&QUERY_NUMBER=1) Homo sapiens chromosome 6 genomic ... [38.2](http://www.ncbi.nlm.nih.gov/blast/Blast.cgi#88998766%2388998766) 7.3

[ref|NW_923073.1|HsCraAADB02_255](http://www.ncbi.nlm.nih.gov/mapview/maps.cgi?maps=blast_set&db=all_contig&na=1&gnl=ref|NW_923073.1|HsCraAADB02_255&gi=88999178&term=88999178%5Bgi%5D&taxid=9606&RID=JWC9C717011&QUERY_NUMBER=1) Homo sapiens chromosome 6 gen... [38.2](http://www.ncbi.nlm.nih.gov/blast/Blast.cgi#88999178%2388999178) 7.3

[ref|NW_922596.1|HsCraAADB02_212](http://www.ncbi.nlm.nih.gov/mapview/maps.cgi?maps=blast_set&db=all_contig&na=1&gnl=ref|NW_922596.1|HsCraAADB02_212&gi=88987703&term=88987703%5Bgi%5D&taxid=9606&RID=JWC9C717011&QUERY_NUMBER=1) Homo sapiens chromosome 5 gen... [38.2](http://www.ncbi.nlm.nih.gov/blast/Blast.cgi#88987703%2388987703) 7.3

[ref|NW_922796.1|HsCraAADB02_230](http://www.ncbi.nlm.nih.gov/mapview/maps.cgi?maps=blast_set&db=all_contig&na=1&gnl=ref|NW_922796.1|HsCraAADB02_230&gi=88990009&term=88990009%5Bgi%5D&taxid=9606&RID=JWC9C717011&QUERY_NUMBER=1) Homo sapiens chromosome 5 gen... [38.2](http://www.ncbi.nlm.nih.gov/blast/Blast.cgi#88990009%2388990009) 7.3

[ref|NW_922784.1|HsCraAADB02_229](http://www.ncbi.nlm.nih.gov/mapview/maps.cgi?maps=blast_set&db=all_contig&na=1&gnl=ref|NW_922784.1|HsCraAADB02_229&gi=88989841&term=88989841%5Bgi%5D&taxid=9606&RID=JWC9C717011&QUERY_NUMBER=1) Homo sapiens chromosome 5 gen... [38.2](http://www.ncbi.nlm.nih.gov/blast/Blast.cgi#88989841%2388989841) 7.3

[ref|NW_922562.1|HsCraAADB02_209](http://www.ncbi.nlm.nih.gov/mapview/maps.cgi?maps=blast_set&db=all_contig&na=1&gnl=ref|NW_922562.1|HsCraAADB02_209&gi=88987555&term=88987555%5Bgi%5D&taxid=9606&RID=JWC9C717011&QUERY_NUMBER=1) Homo sapiens chromosome 5 gen... [38.2](http://www.ncbi.nlm.nih.gov/blast/Blast.cgi#88987555%2388987555) 7.3

[ref|NW_922751.1|HsCraAADB02_226](http://www.ncbi.nlm.nih.gov/mapview/maps.cgi?maps=blast_set&db=all_contig&na=1&gnl=ref|NW_922751.1|HsCraAADB02_226&gi=88988928&term=88988928%5Bgi%5D&taxid=9606&RID=JWC9C717011&QUERY_NUMBER=1) Homo sapiens chromosome 5 gen... [38.2](http://www.ncbi.nlm.nih.gov/blast/Blast.cgi#88988928%2388988928) 7.3

[ref|NW_922729.1|HsCraAADB02_224](http://www.ncbi.nlm.nih.gov/mapview/maps.cgi?maps=blast_set&db=all_contig&na=1&gnl=ref|NW_922729.1|HsCraAADB02_224&gi=88988511&term=88988511%5Bgi%5D&taxid=9606&RID=JWC9C717011&QUERY_NUMBER=1) Homo sapiens chromosome 5 gen... [38.2](http://www.ncbi.nlm.nih.gov/blast/Blast.cgi#88988511%2388988511) 7.3

[ref|NW_922217.1|HsCraAADB02_178](http://www.ncbi.nlm.nih.gov/mapview/maps.cgi?maps=blast_set&db=all_contig&na=1&gnl=ref|NW_922217.1|HsCraAADB02_178&gi=88981534&term=88981534%5Bgi%5D&taxid=9606&RID=JWC9C717011&QUERY_NUMBER=1) Homo sapiens chromosome 4 gen... [38.2](http://www.ncbi.nlm.nih.gov/blast/Blast.cgi#88981534%2388981534) 7.3

[ref|NW_922073.1|HsCraAADB02_165](http://www.ncbi.nlm.nih.gov/mapview/maps.cgi?maps=blast_set&db=all_contig&na=1&gnl=ref|NW_922073.1|HsCraAADB02_165&gi=88979823&term=88979823%5Bgi%5D&taxid=9606&RID=JWC9C717011&QUERY_NUMBER=1) Homo sapiens chromosome 4 gen... [38.2](http://www.ncbi.nlm.nih.gov/blast/Blast.cgi#88979823%2388979823) 7.3

[ref|NW_922162.1|HsCraAADB02_173](http://www.ncbi.nlm.nih.gov/mapview/maps.cgi?maps=blast_set&db=all_contig&na=1&gnl=ref|NW_922162.1|HsCraAADB02_173&gi=88980670&term=88980670%5Bgi%5D&taxid=9606&RID=JWC9C717011&QUERY_NUMBER=1) Homo sapiens chromosome 4 gen... [38.2](http://www.ncbi.nlm.nih.gov/blast/Blast.cgi#88980670%2388980670) 7.3

[ref|NW_921651.1|HsCraAADB02_127](http://www.ncbi.nlm.nih.gov/mapview/maps.cgi?maps=blast_set&db=all_contig&na=1&gnl=ref|NW_921651.1|HsCraAADB02_127&gi=88969293&term=88969293%5Bgi%5D&taxid=9606&RID=JWC9C717011&QUERY_NUMBER=1) Homo sapiens chromosome 3 gen... [38.2](http://www.ncbi.nlm.nih.gov/blast/Blast.cgi#88969293%2388969293) 7.3

[ref|NW_921807.1|HsCraAADB02_141](http://www.ncbi.nlm.nih.gov/mapview/maps.cgi?maps=blast_set&db=all_contig&na=1&gnl=ref|NW_921807.1|HsCraAADB02_141&gi=88971630&term=88971630%5Bgi%5D&taxid=9606&RID=JWC9C717011&QUERY_NUMBER=1) Homo sapiens chromosome 3 gen... [38.2](http://www.ncbi.nlm.nih.gov/blast/Blast.cgi#88971630%2388971630) 7.3

[ref|NW_921585.1|HsCraAADB02_121](http://www.ncbi.nlm.nih.gov/mapview/maps.cgi?maps=blast_set&db=all_contig&na=1&gnl=ref|NW_921585.1|HsCraAADB02_121&gi=88958353&term=88958353%5Bgi%5D&taxid=9606&RID=JWC9C717011&QUERY_NUMBER=1) Homo sapiens chromosome 2 gen... [38.2](http://www.ncbi.nlm.nih.gov/blast/Blast.cgi#88958353%2388958353) 7.3

[ref|NW_927719.1|HsCraAADB02_69](http://www.ncbi.nlm.nih.gov/mapview/maps.cgi?maps=blast_set&db=all_contig&na=1&gnl=ref|NW_927719.1|HsCraAADB02_69&gi=88955854&term=88955854%5Bgi%5D&taxid=9606&RID=JWC9C717011&QUERY_NUMBER=1) Homo sapiens chromosome 2 geno... [38.2](http://www.ncbi.nlm.nih.gov/blast/Blast.cgi#88955854%2388955854) 7.3

[ref|NW_921795.1|HsCraAADB02_14](http://www.ncbi.nlm.nih.gov/mapview/maps.cgi?maps=blast_set&db=all_contig&na=1&gnl=ref|NW_921795.1|HsCraAADB02_14&gi=88951910&term=88951910%5Bgi%5D&taxid=9606&RID=JWC9C717011&QUERY_NUMBER=1) Homo sapiens chromosome 1 geno... [38.2](http://www.ncbi.nlm.nih.gov/blast/Blast.cgi#88951910%2388951910) 7.3

[ref|NW_921351.1|HsCraAADB02_10](http://www.ncbi.nlm.nih.gov/mapview/maps.cgi?maps=blast_set&db=all_contig&na=1&gnl=ref|NW_921351.1|HsCraAADB02_10&gi=88950243&term=88950243%5Bgi%5D&taxid=9606&RID=JWC9C717011&QUERY_NUMBER=1) Homo sapiens chromosome 1 geno... [38.2](http://www.ncbi.nlm.nih.gov/blast/Blast.cgi#88950243%2388950243) 7.3

[ref|NW_927128.1|HsCraAADB02_62](http://www.ncbi.nlm.nih.gov/mapview/maps.cgi?maps=blast_set&db=all_contig&na=1&gnl=ref|NW_927128.1|HsCraAADB02_62&gi=88952908&term=88952908%5Bgi%5D&taxid=9606&RID=JWC9C717011&QUERY_NUMBER=1) Homo sapiens chromosome 1 geno... [38.2](http://www.ncbi.nlm.nih.gov/blast/Blast.cgi#88952908%2388952908) 7.3

[ref|NW_922462.1|HsCraAADB02_20](http://www.ncbi.nlm.nih.gov/mapview/maps.cgi?maps=blast_set&db=all_contig&na=1&gnl=ref|NW_922462.1|HsCraAADB02_20&gi=88952330&term=88952330%5Bgi%5D&taxid=9606&RID=JWC9C717011&QUERY_NUMBER=1) Homo sapiens chromosome 1 geno... [38.2](http://www.ncbi.nlm.nih.gov/blast/Blast.cgi#88952330%2388952330) 7.3
